# Simvastatin protects ischemic spinal cord injury from cell death and cytotoxicity through decreasing oxidative stress: in vitro primary cultured rat spinal cord model under oxygen and glucose deprivation-reoxygenation conditions

**DOI:** 10.1186/s13018-017-0536-9

**Published:** 2017-02-27

**Authors:** Hye-Min Sohn, Jin-Young Hwang, Jung-Hee Ryu, Jinhee Kim, Seongjoo Park, Jin-woo Park, Sung-Hee Han

**Affiliations:** 1 Department of Anesthesiology and Pain Medicine, Seoul National University Bundang Hospital, 82 Gumi-ro, 173 Beon-gil, Bundang-gu, Seongnam-si, Gyeonggi-do 13620 Republic of Korea; 2 Department of Anesthesiology and Pain Medicine, SMG-SNU Boramae Medical Center, Seoul, Republic of Korea

**Keywords:** Ischemia–reperfusion injury, Neuroprotection, Oxidative stress, Oxygen–glucose deprivation, Simvastatin, Spinal cord injury

## Abstract

**Background:**

Ischemia and the following reperfusion damage are critical mechanisms of spinal cord injury. Statins have been reported to decrease ischemia–reperfusion injury in many organs including the spinal cord. Anti-oxidative effect is one of the main protective mechanisms of statin against neuronal death and cytotoxicity. We hypothesized that statins’ anti-oxidative property would yield neuroprotective effects on spinal cord ischemia–reperfusion injury

**Methods:**

Primary cultured spinal cord motor neurons were isolated from Sprague–Dawley rat fetuses. Ischemia–reperfusion injury model was induced by 60 min of oxygen and glucose deprivation (OGD) and 24 h of reoxygenation. Healthy and OGD cells were treated with simvastatin at concentrations of 0.1, 1, and 10 μM for 24 h. Cell viability was assessed using water-soluble tetrazolium salt (WST)-8, cytotoxicity with LDH, and production of free radicals with DCFDA (2′,7′-dichlorofluorescein diacetate).

**Results:**

OGD reduced neuronal viability compared to normoxic control by 35.3%; however, 0.1–10 μM of simvastatin treatment following OGD improved cell survival. OGD increased LDH release up to 214%; however, simvastatin treatment attenuated its cytotoxicity at concentrations of 0.1–10 μM (*p* < 0.001 and *p* = 0.001). Simvastatin also reduced deteriorated morphological changes of motor neurons following OGD. Oxidative stress was reduced by simvastatin (0.1–10 μM) compared to untreated cells exposed to OGD (*p* < 0.001).

**Conclusions:**

Simvastatin effectively reduced spinal cord neuronal death and cytotoxicity against ischemia–reperfusion injury, probably via modification of oxidative stress.

## Background

Spinal cord injury (SCI) can lead to devastating complications, including permanent neurological damage [1–3]. In acute traumatic spinal cord injury, ischemia and the following reperfusion play a critical role in primary mechanical and secondary pathophysiological mechanisms [4–6]. After the initial rapid compression and trauma, spinal cord ischemia occurs via various mechanisms, including direct injury to the microvasculature, reduced spinal cord blood flow, and disrupted spinal cord autoregulation [5]. In the following passages, restoration of vascular perfusion and surgical intervention are an essential part of the treatment approach to avoid persistent compression; however, reperfusion *per se* can cause further damage [7–9].

Spinal cord ischemia–reperfusion damage is also an important cause of postoperative neurological deficits following decompression surgery, which are rare but very serious. For example, 1.5–6.3% of patients with cervical spondylotic myelopathy suffer postoperative delayed paraplegia related to ischemia–reperfusion injury of the spinal cord [7–9]. In such cases, neuroprotection against ischemia–reperfusion is crucial to prevent spinal cord injury.

Statins, 3-hydroxy-3-methyl-glutaryl-coenzyme A (HMG-CoA) reductase inhibitors, have been shown to minimize the severity of ischemia–reperfusion injury in many organs including the brain, heart, kidney, and lung [10–14]. Statins attenuate neuronal injury and promote neurologic recovery after cerebral ischemia in experimental animal models and in vitro cellular models [15–18]. Statins are frequently used as cholesterol-lowering agents, but their protective effect against ischemia depends on other actions as well [19], including modification of oxidative stress [16, 20–22], anti-inflammatory effects, and immunomodulation [15, 18].

Statins have been repeatedly reported to be neuroprotective against spinal cord injury, demonstrating neurologic and histopathologic improvements [23–27]. Especially simvastatin, since it readily crosses the blood–spinal cord barrier, could be widely used to treat spinal cord injuries in clinical practice [24]. As yet, the underlying mechanism has not been fully studied. In models of cerebral ischemia, simvastatin attenuated neuronal death by reducing the production and toxicity of oxidative stress-related markers [28, 29]. However, statins’ beneficial antioxidant properties in spinal cord neurons have not yet been investigated.

In this study, we sought the efficacy of simvastatin in attenuation of SCI-induced pathology. We first demonstrated that ischemia–reperfusion injury elicits motor neuron death and cytotoxicity in this model of SCI, and then investigated whether simvastatin treatment recovers those deteriorations of spinal cord neurons against oxidative stress as its neuroprotective mechanism of action.

## Methods

### Primary culture of spinal cord neuron

The animal procedures were carried out in Seoul National University Bundang Hospital according to an approved animal research protocol (IRB number 63-13-034). Timed-pregnant Sprague–Dawley rats were obtained, and primary rat spinal cord neurons were isolated from embryonic day 14–15 rat fetuses using a previously described method [30]. Briefly, embryonic vertebral canals were opened, meninges and blood vessels were cleared away using sterile fine-tipped forceps, and the embryonic spinal cords were sliced into small pieces using a scalpel. After microdissection and trituration, the isolated cells were seeded on poly-l-lysine (200 μg/mL) (PLL) (Sigma-Aldrich, St. Louis, MO, USA) coated plates at a concentration of 10^5^ cells/well and maintained in a 5% CO_2_ incubator at 37 °C. Cells were cultured in neurobasal medium (Gibco, Carlsbad, CA, USA) supplemented with 2% B27 supplement (Gibco) and 2 mM glutamine (Gibco). After 3 days in vitro (DIV), 5 μM cytosine-β-d-arabinofuranoside (AraC) (Sigma-Aldrich, St. Louis, MO, USA) was added into the medium to inhibit non-neuronal cell proliferation. One half of the culture medium was replaced by a fresh medium every 3 days.

### Oxygen and glucose deprivation (OGD) followed by reoxygenation

OGD and reoxygenation were carried out in cultures after 7 DIV as described previously [31]. Briefly, on the seventh day, the original media was removed and replaced with glucose-free DMEM. The cultures were then transferred to an anaerobic incubator containing a mixture of 95% N_2_ and 5% CO_2_ at 37 °C. Several pilot experiments with various durations of OGD and reoxygenation indicated that 60 min of OGD and 24-h recovery led to sufficient injury for this study.

### Treatment with simvastatin

Simvastatin (Sigma-Aldrich, St Louis, MO, USA) of 4 mg was dissolved in 100 μL of ethanol, with subsequent addition of 150 μL of 0.1 N NaOH. This solution was incubated at 50 °C for 2 h and then neutralized with HCl to pH 7. The resulting solution had a final volume of 1 mL with sterile phosphate-buffered saline [32]. To examine the toxicity of simvastatin on motor neurons, various concentrations of simvastatin (0.1–50 μM) were applied to healthy motor neurons for 24 h. In the main experiment, simvastatin was applied to the ischemia–reperfusion-injured motor neurons at concentrations of 0, 0.1, 1, and 10 μM for 24 h. Injured control cultures (OGD only) were given equal volume of phosphate-buffered vehicle. Each dose of simvastatin was applied from the start of the OGD and was maintained during the following 24-h reoxygenation period.

### Determination of cellular viability

Cellular viability was assessed with tetrazolium salt reduction assay. This is a colorimetric method for determining the number of living cells. The viable cells containing NADH or NADPH can convert tetrazolium compound, WST-8, 2-(2-methoxy-4-nitrophenyl)-3-(4-nitrophenyl)-5-(2,4-disulfophenyl)-2H tetrazolium, into formazan product that is soluble in tissue culture medium [33, 34]. According to the manufacturer’s instruction (cell counting kit 8 (CCK-8), Sigma-Aldrich, St. Louis, MO, USA), 10 μL of WST-8 solution was added to each well in 96-well plates. After 2-h incubation at 37 °C, absorbance at 450 nm was measured using a microplate reader. The quantity of formazan product is directly proportional to the number of living cells in the culture, and the results are expressed as the percentage of viable cells relative to untreated controls.

### Determination of cytotoxicity

Lactate dehydrogenase (LDH) is a stable cytoplasmic enzyme that is released into the cell culture supernatant when the cytoplasmic membrane is ruptured [28, 35, 36]. LDH activity in the medium was determined using a colorimetric diagnostic kit (CytoTox96^®^ Non-Radioactive Cytotoxicity Assay, Promega, Madison, WI, USA). In brief, 50-μL aliquots of the culture medium and 50 μL of reconstituted substrate were mixed and incubated for 30 min at 37 °C in the dark, after which the reaction was terminated with a stop solution (1 M HCl, 50 μL). The color intensity is directly proportional to the number of lysed cells and can be qualified by reading the absorbance at 490 nm. The percentage of cytotoxicity is determined by calculating the optical density at 490 nm (OD_490nm_) and subtracting from the absorbance value obtained in the background control.

### Measurement of free radical production

The production of free radicals was assessed by using DCFDA (2′,7′-dichlorofluorescein diacetate), which is one of the most widely used techniques for direct measuring of cell redox state [37]. DCFDA is a fluorogenic dye for highly selective detection of hydroxyl, peroxyl, and any other intracellular reactive oxygen species (ROS) activity. DCFDA is diffused into cells and is deacetylated by cellular esterases into a non-fluorescent compound that is subsequently oxidized by ROS into DCF (2′,7′-dichlorofluorescein). DCF is a highly fluorescent compound and hence is detectable by fluorescence spectroscopy.

The DCFDA assay was performed according to the manufacturer’s instruction (DCFDA Cellular ROS Detection Kit, Abcam, Cambridge, UK). In brief, 10 μL of DCFDA was added to cells and incubated for 30 min. After being washed out with PBS, the intensity of fluorescence was examined by flow cytometry. Accumulation of the oxidized fluorescent derivate (DCF) in the cells was measured at emission and excitation wavelengths of 530 and 485 nm, respectively.

### Statistical analysis

Data are expressed as mean ± SEM. Statistical comparisons between groups were done using *t* tests, and comparisons between three or more groups were performed by one-way ANOVA followed by Dunnett T3 post hoc comparisons. A *p* value less than 0.05 was considered significant. All statistical analyses were performed using SPSS 19.0 software for Windows (SPSS, Chicago, IL, USA).

## Results

### Motoneuron cultures and effects of simvastatin on healthy motor neuron

**Fig. 1:**
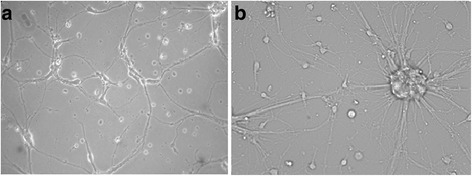
Typical morphology of healthy primary spinal cord neurons from rats. **a** The healthy neurons showed extensive neurite outgrowth and neurons with a dense meshwork on day 4. **b** Much denser meshwork can be seen on day 7 compared to (**a**)

After 7 to 8 days in culture, cells showed neuronal morphological characteristics and an extensive meshwork of neurite outgrowth (Fig. 1a, b).

**Fig. 2:**
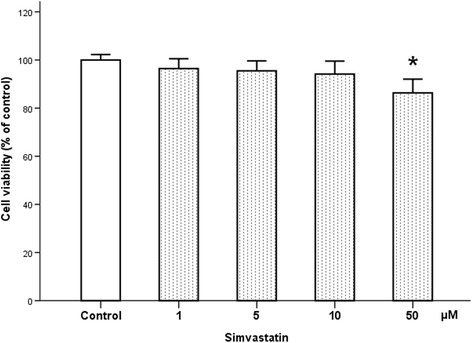
Effect of simvastatin on the viability of healthy motor neuron. Effect of simvastatin on the viability of motor neuron was assessed with WST-8 assay. There were no differences in the viability of healthy cells depending on the concentration of simvastatin. Simvastatin showed little or no effect on normal motoneuron ranges from 1 to 10 μM. However, at concentration of 50 μM, WST-8 statistically decreased compared to control cell. Data are expressed as the percentage of total WST-8 and represent the mean ± SEM. *Asterisk* denotes significant difference from control (*n* = 16; *p* < 0.05)

Concentrations of simvastatin from 1 to 50 μM were applied to healthy control cells. Concentrations of 1, 5, and 10 μM did not affect the viability of cultured spinal neurons as indicated by WST-8. In contrast, at a concentration of 50 μM, cell viability was statistically decreased compared to the control cells (*p* = 0.002). Since the 50 μM concentration seemed to be toxic, we applied simvastatin at concentrations of 1 to 10 μM in further experiments (Fig. 2).

### The effect of simvastatin on ischemia–reperfusion-induced cell death and cytotoxicity

**Fig. 3:**
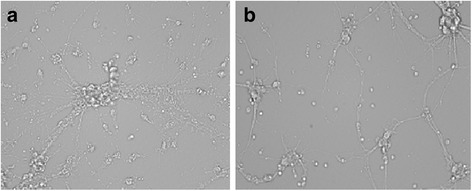
Morphological change in the motor neuron following ischemia–reperfusion injury with or without simvastatin. **a** Without simvastatin. Ischemia–reperfusion damage resulted in massive cell loss and destruction of neural networks. **b** With simvastatin. Treatment of simvastatin markedly increased neuronal survival after ischemia–reperfusion damage. Cells treated with simvastatin showed preserved morphological features of neurons with a denser meshwork of neurites when compared to (**a**)

Spinal cord ischemia–reperfusion injury was simulated by 60 min of OGD and 24 h of reoxygenation. This duration of injury resulted in moderate loss of the neural meshwork and morphological changes in the remaining cells (Fig. 3).

**Fig. 4:**
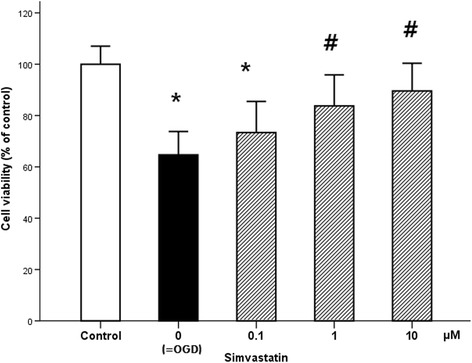
Effect of simvastatin on the viability of the motor neuron following ischemia–reperfusion injury. Effect of OGD and simvastatin on the viability of motor neuron was assessed with WST-8 assay. OGD resulted in marked reduction of cellular viability. Simvastatin, treated at the indicated concentrations, resulted in an increase in cell survival, particularly at 1 and 10 μM. Values are represented as means ± SEM; *asterisk* denotes significant difference from control. *Number sign* denotes significant difference from the OGD cells (*n* = 16; *p* < 0.05). *OGD* oxygen and glucose deprivation

The ischemia–reperfusion injury dramatically reduced cell survival (35% decrease) measured by WST-8 assay compared to the control culture. Simvastatin markedly ameliorated OGD/reoxygenation-evoked cell death at concentrations of 1 and 10 μM (*p* = 0.032 and *p* = 0.008, respectively) when compared to injured controls (Fig. 4). The protective effect was shown within a range of 0.1 to 10 μM. However, simvastatin did not fully restore viability of ischemia–reperfusion-injured motor neurons to that of healthy controls.

**Fig. 5:**
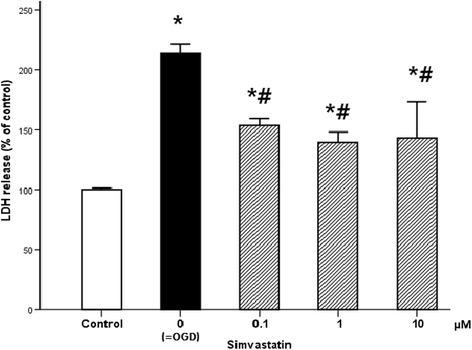
Effect of simvastatin on the cytotoxicity following ischemia–reperfusion injury. Cytotoxicity of OGD was assessed with LDH release. Cells were subjected to 60 min of OGD and 24 h of recovery time. LDH significantly increased after OGD compared to healthy cells. Simvastatin treatment (0.1, 1, and 10 μM) effectively attenuated LDH release. Values are represented as means ± SEM; *asterisk* denotes significant difference from control. *Number sign* denotes significant difference from the OGD cells (*n* = 16; *p* < 0.05). *LDH* lactate dehydrogenase, *OGD* oxygen and glucose deprivation

The effects of simvastatin on the motor neuron damage are shown in Fig. 5. The ischemia–reperfusion damage resulted in cell death, as evidenced by an increase in LDH (214%). Within a range of 0.1 to 10 μM, simvastatin-treated motor neurons showed significantly lower values for LDH when compared to injured controls (*p* < 0.001 and *p* = 0.001, respectively), though cell death was not completely blocked.

### The effect of simvastatin on ischemia–reperfusion-induced oxidative stress

**Fig. 6:**
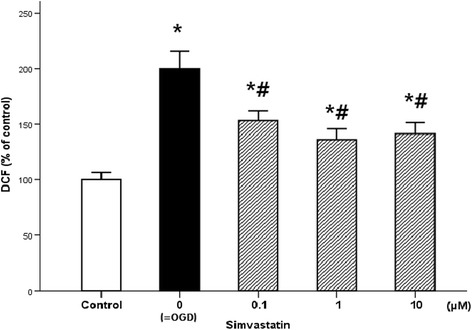
Effect of simvastatin on the motor neuron oxidative stress induced with ischemia–reperfusion. Oxidative stress of the motor neuron was assessed with DCFDA assay. OGD led to marked increase in DCF labeling of cells. Simvastatin treatment (0.1, 1, and 10 μM) effectively reduced DCF labeling following OGD. Values are represented as means ± SEM; *asterisk* denotes difference from control, and *number sign* denotes difference from OGD cells (*n* = 16; *p* < 0.05). *OGD* oxygen and glucose deprivation, *DCFDA* 2′,7′-dichlorofluorescein diacetate, *DCF* 2′,7′-dichlorofluorescein

OGD and reoxygenation dramatically increased DCFDA value, implying higher levels of oxidative stress. Simvastatin effectively reduced the oxidative stress. All tested doses of simvastatin (0.1–10 μM) attenuated the rise in DCFDA level (*p* < 0.001 at all experimental concentrations), but not to a value as low as that of the healthy controls (Fig. 6).

## Discussion

The present study demonstrated that motor neuron death, cytotoxicity, and oxidative stress induced by ischemia–reperfusion injury are significantly attenuated by simvastatin. To our knowledge, this is the first study to suggest the protective role of statins against ischemic injury of spinal cord neurons devising a primary motor neuron culture.

OGD and reoxygenation are well established and reliable neuronal cellular injury models that mimic changes that occur after ischemic insult in vivo [38–40]. Neurons are the cell type most sensitive to ischemic injury. Previous studies of cerebral ischemia–reperfusion have demonstrated that ischemia–reperfusion injury leads to obvious morphological neuronal changes, decreased cell survival, and dramatic increases in LDH release [38–40]. Our model of motor neuron ischemia–reperfusion yielded similar damages; motor neurons of primary cultured rat spinal cord resulted in a significant decrease in cell viability as evidenced by WST-8. After OGD, almost twice as much LDH had leaked out through the injured cell membrane as was leaked from healthy cells, implying increased neurotoxicity. Also OGD and reoxygenation dramatically increased DCFDA value, implying enhanced free radical generation in cells.

Statins reduce OGD and reoxygenation-induced neuronal injury [28, 40]. Statins reduce cerebral infarct volume [41, 42], improve perfusion deficits [43], and facilitate cognitive improvement [17] mostly using the experimental preclinical stroke models [39, 43]. Protective effect of statins against ischemic injury has also been reported in other organs, such as myocardial ischemia–reperfusion injury [11, 44], ischemic acute kidney injury [14], and intestinal ischemia–reperfusion injury [45]. We obtained similar results; simvastatin effectively attenuated ischemia–reperfusion-induced spinal cord motor neuron death. Quantitative analysis showed an increase in cell survival after simvastatin treatment at concentrations from 0.1 to 10 μM.

Similarly, ischemia–reperfusion-evoked LDH release was reduced by simvastatin in a dose-dependent manner, which supports the protective effect of simvastatin against ischemia–reperfusion-induced cytotoxicity. LDH leakage indicates cytotoxicity as a result of cell membrane disintegration [20]. The elevated LDH following OGD in our study significantly decreased with simvastatin treatment at concentrations of 0.1, 1, and 10 μM, indicating that it attenuates the cellular injury/death induced by ischemia. Our basic research performed at the cellular level demonstrated the safety margin of simvastatin in terms of motor neuron protection.

Ischemic injury leads to production of massive amounts of ROS that directly damage the main cellular constituents [18, 22]. In addition, reperfusion to an ischemic organ, a restoration of oxygen levels in hypoxic tissues, also stimulates ROS production [46, 47]. However, the CNS is extremely sensitive to oxidative stress due to delicate lipid layers of its cell membranes and low levels of antioxidant enzymes [48]. Oxidative stress responses in the CNS vary among different cell types. Neurons have relatively low antioxidant capacity and limited scope to upregulate it upon increased oxidative stress, so they are much more vulnerable to oxidative damage than other cells in CNS [49, 50]. Previous studies investigated the correlation between increased production of ROS and neuronal death following ischemia/hypoxia [51, 52]. Simvastatin has been reported to have pleiotropic effects, including reducing oxidative stress [18, 19, 53].

In our study, simvastatin significantly decreased free radical production induced by ischemia–reperfusion, implying that the drug modifies ischemia–reperfusion-induced oxidative stress. Numerous studies have reported that statins decrease ROS production in tissue and in vivo models and in cultured neurons derived from embryonic rat brain tissue [18, 20, 29] and following major vascular surgery [54] including thoracoabdominal aneurysm repair [55, 56] and decompression surgery of degenerative changes of the spine including cervical spondylotic myelopathy [57–59]. Our study results agree with these findings, showing that simvastatin reduces the production of DCFDA in spinal cord cells exposed to OGD and reoxygenation. DCFDA is one of the most widely used techniques for directly measuring the redox state of a cell [60].

Despite numerous reports of the neuroprotective effects of statins [19, 28, 29, 61–63], some investigators have reported that they are toxic to neurons in vitro [64, 65]. The toxicity versus protective effects of statins are dependent upon various factors, including the pharmacological characteristics of the individual statin agent, concentration of the drug, and cholesterol content of the neural cell used in the experiment [66]. Therefore, before the main experiment, we evaluated simvastatin’s toxicity to healthy motor neurons using concentrations ranging from 0.1 to 50 μM, based on the previous studies [28, 66–70]. Cellular viability, assessed by WST-8, was not affected at any experimental simvastatin dose from 0.1 to 10 μM. However, with a dose of 50 μM, the viability of the normal motor neuron was significantly reduced. This finding is consistent with that of previous reports and suggests possible cytotoxicity of high-dose statins [71]; that high dose was not applied in the main experiments.

The limitation of this study is that we did not determine the precise mechanism of action of simvastatin. Our capabilities did not allow us to examine whether the protective effects were due to direct reduction of cholesterol or whether indirect/nonspecific mechanisms of the statins modify cellular signaling. Further studies will help to elucidate the exact mechanism and the long-term effects of statins for clinical application. Second, there were no additional experiments to show antagonizing the neuroprotective effect of simvastatin, by coapplication of other drugs, such as mevalonate, one of the downstream products of HMG-CoA reductase. Third, it is significant that our spinal cord cells were cultured from embryonic 14–15 fetus because only embryonic cells can be used for extended culture. However, younger cells are more sensitive to neurotoxins, and therefore, the results should generalize to adults with caution.

## Conclusions

In conclusion, simvastatin reduces ischemia and reperfusion-induced injury of spinal cord motor neurons through its antioxidant effects. Our results could lead to clinical use of simvastatin to treat this type of spinal cord injury.

## Abbreviations

DCFDA: 2′,7′-dichlorofluorescein diacetate
DIV: Days in vitro
LDH: Lactate dehydrogenase
OGD: Oxygen and glucose deprivation
ROS: Reactive oxygen species
SCI: Spinal cord injury
WST: Water-soluble tetrazolium salt
